# Molecular Mechanisms of Leukocyte Migration and Its Potential Targeting—Lessons Learned From MKL1/SRF-Related Primary Immunodeficiency Diseases

**DOI:** 10.3389/fimmu.2021.615477

**Published:** 2021-02-22

**Authors:** Evelien G. G. Sprenkeler, Carla Guenther, Imrul Faisal, Taco W. Kuijpers, Susanna C. Fagerholm

**Affiliations:** ^1^ Department of Blood Cell Research, Sanquin Research, Amsterdam University Medical Center (AUMC), University of Amsterdam, Amsterdam, Netherlands; ^2^ Department of Pediatric Immunology, Rheumatology, and Infectious Diseases, Emma Children’s Hospital, Amsterdam University Medical Center (AUMC), University of Amsterdam, Amsterdam, Netherlands; ^3^ Molecular and Integrative Biosciences Research Programme, Faculty of Biological and Environmental Sciences, University of Helsinki, Helsinki, Finland

**Keywords:** MKL1, SRF, neutrophil, migration, immunodeficiency—primary

## Abstract

Megakaryoblastic leukemia 1 (MKL1) deficiency is one of the most recently discovered primary immunodeficiencies (PIDs) caused by cytoskeletal abnormalities. These immunological “actinopathies” primarily affect hematopoietic cells, resulting in defects in both the innate immune system (phagocyte defects) and adaptive immune system (T-cell and B-cell defects). MKL1 is a transcriptional coactivator that operates together with serum response factor (SRF) to regulate gene transcription. The MKL/SRF pathway has been originally described to have important functions in actin regulation in cells. Recent results indicate that MKL1 also has very important roles in immune cells, and that MKL1 deficiency results in an immunodeficiency affecting the migration and function of primarily myeloid cells such as neutrophils. Interestingly, several actinopathies are caused by mutations in genes which are recognized MKL(1/2)-dependent SRF-target genes, namely *ACTB*, *WIPF1*, *WDR1*, and *MSN*. Here we summarize these and related (ARPC1B) actinopathies and their effects on immune cell function, especially focusing on their effects on leukocyte adhesion and migration. Furthermore, we summarize recent therapeutic efforts targeting the MKL/SRF pathway in disease.

## Introduction

Leukocytes constantly traffic between different compartments in the body, and need to be able to employ different types of adhesion and migration modes in different types of environments. Briefly, leukocytes adhere to endothelial cells under shear flow conditions using adhesion receptors such as selectins, integrins, and intercellular adhesion molecules, utilize 2D migration modes on the endothelial cell layer, and migrate in complex tissue environments utilizing 3D migration. Whereas 2D migration is strongly dependent on cell adhesion (i.e., ligand binding *via* integrins), the 3D amoeboid migration mode of leukocytes such as dendritic cells is associated with very low adhesion strength (i.e., integrin-independent) and relies on cytoskeletal deformation instead (1, 2).

Both during 2D and 3D migration, actin dynamics in migrating cells is complex and regulated by both positive and negative regulators. Actomyosin contraction at the cell rear end (uropod) aids in moving the cytoplasm and cell body, while the actin-related protein (ARP) 2/3 complex is an important mediator of actin polymerization at the leading edge (1). The small GTPases Rac and Cdc42 localize to the leading edge and regulate actin polymerization (2). In contrast, RhoA localizes to the uropod where it regulates actin cables (through the formin mDia) and actomyosin contractility (2). Proteins binding to G-actin, such as ADF/cofilin, and proteins severing and capping actin filaments, such as gelsolin, are also important factors regulating actin dynamics in migrating cells (3).

Primary immunodeficiencies (PIDs) are rare genetic disorders of the immune system, and lead to immune deficiencies of various severity. By studying primary immunodeficiencies, much has been learned about the molecular basis of immune system function, including leukocyte trafficking. In a classical example, Wiskott-Aldrich syndrome is caused by defects in the WAS protein (WASP), which plays essential roles in the regulation of the actin cytoskeleton upon cell activation. Consequently, WASP deficiency leads to fundamental defects of the immune system, including leukocyte migration, as well as a platelet defect with bleeding tendency because of reduced platelet counts (4).

Leukocyte adhesion deficiency type I-II and -III in turn are caused by defects in beta2-integrins, selectins, and kindlin-3, respectively, and result in severe defects in leukocyte (especially neutrophil) trafficking into sites of inflammation, as well as specific defects in adaptive immunity (5). Notably, all abovementioned defects (apart from LAD-II, which is a metabolic disorder which also affects leukocytes and neutrophil extravasation) represent typical hematopoietic disorders. This is explained by the lack of protein expression outside of the hematopoietic system and/or lack of redundancy in activity by homologous proteins that may substitute for the protein that is lacking or dysfunctional in activity. The selective expression of these proteins within the hematopoietic system also explains that curative treatment currently (still) consists of bone marrow transplantation.

Megakaryoblastic leukemia 1 (MKL1) deficiency is one of the most recently identified primary immunodeficiencies that causes a rare defect in actin-dependent processes, including leukocyte adhesion and migration. Here, we review what is known about MKL1 deficiency and other MKL/SRF (serum response factor)-related actin-based primary immunodeficiencies. In this review, we will focus on the proteins involved in this major pathway, their roles in immune cell migration and effector functions, and discuss potential lessons to be learned from these diseases and new opportunities with regards to therapeutic targeting of this pathway.

## The MKL1/SRF Pathway

MKL1, also called MRTF-A (myocardin-related transcription factor-A) or MAL (megakaryocytic acute leukemia), is a transcriptional co-regulator expressed in many cell types. It has long been known to have important roles in regulating actin and other cytoskeleton genes in many types of cells, together with the transcription factor SRF. There are two isoforms of MKL, MKL1, and MKL2, which have similar roles in cells. However, they also have non-redundant roles, as MKL2 knockout mice are embryonic lethal (6), while MKL1 knockout mice have a milder phenotype. MKL1 knockout mice show partial embryonic lethality, abnormal mammary gland function and reduced platelet count (7).

**Figure 1:**
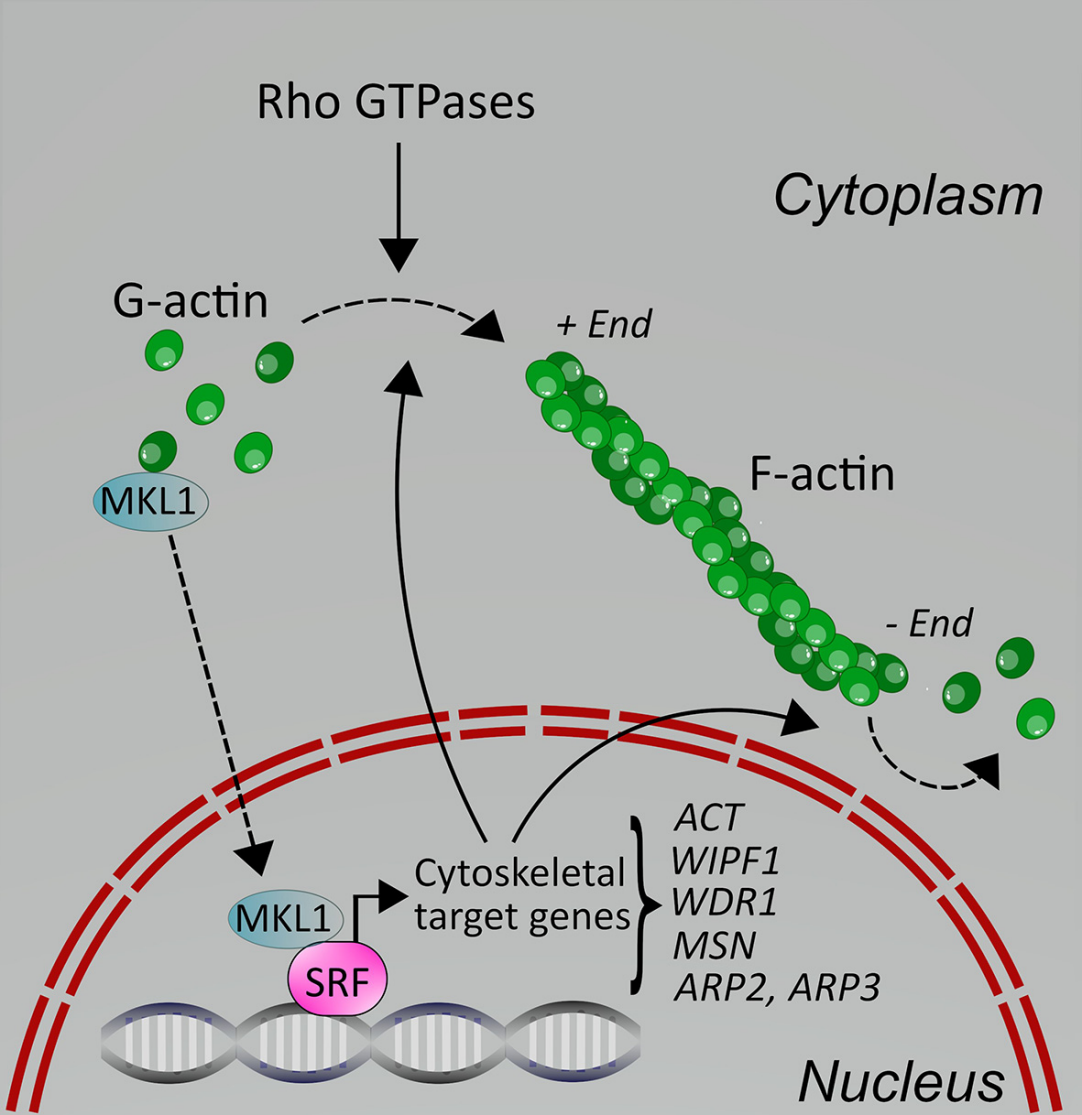
Schematic depicting the molecular regulation of MKL1 in cells. In resting cells, MKL1 is sequestered in the cytoplasm due to binding to G-actin through its RPEL motifs. Upon cell activation through various stimuli, including serum, chemokines and integrins, Rho GTPases are activated, leading to F-actin polymerization. This releases MKL1, allowing it to translocate into the nucleus where it can activate gene transcription together with SRF. Some of the cytoskeletal target genes are shown here; for additional details, see text. Cytoskeletal target genes impact on the actin cytoskeleton in cells, leading to changes in processes such as cell adhesion and motility.

**Figure 2:**
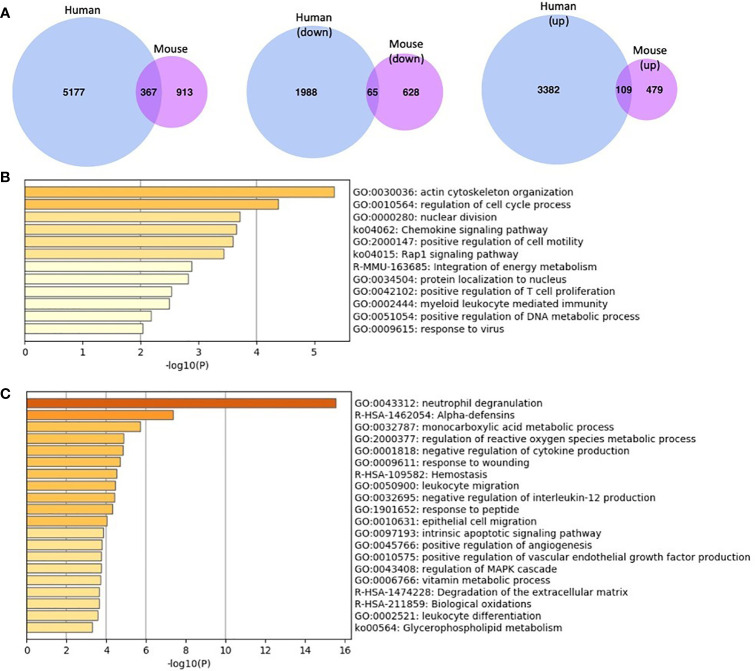
Comparison of gene expression profiles for MKL1-deficient leukocytes (human and murine cells) **(A)** Differentially expressed genes (both up- and downregulated) in MKL1-deficient cells. There were 367 genes in common between them. The second and third Venn diagrams illustrate differentially expressed genes according to expression profile, i.e., upregulated and downregulated separately. There were 65 common downregulated genes and 109 upregulated genes between human and mouse MKL1 deficient cells. The mouse genes were converted to humans by Ensembl’s BioMart package and then compared to the human genes. The Venn diagrams were created using venn.diagram package in R. **(B, C)** Pathway enrichment analysis using downregulated **(B)** and upregulated **(C)** common genes between human and mouse MKL1-deficient cells. This analysis was performed using Metascape.

MKL1 is an interesting transcriptional coactivator, which is itself regulated by actin cytoskeletal dynamics (8). MKL1 is normally found in the cytoplasm, where it is bound to G-actin, and therefore excluded from the nucleus, which means it cannot regulate gene transcription. When cells receive an activating stimulus, such as serum stimulation, chemokine stimulation or other types of stimuli, RhoA is activated, leading to actin polymerization into F-actin. As a consequence, MKL1 is released from G-actin and transported into the nucleus (**Figure 1**). There, it encounters the transcription factor SRF and together the complex initiates gene transcription. In fact, SRF recruits two families of coactivators, the MKLs and the TCFs (ternary complex factors), to couple gene transcription to growth factor signaling. The MKL/SRF complex is involved in regulating cytoskeletal genes, such as actin, in many types of cells, including leukocytes, thereby influencing actin-dependent processes in cells (**Figure 1**). In macrophages, the SRF pathway indeed regulates the expression of cytoskeletal genes (9). In B cells, however, deletion of SRF also led to decreased expression of IgM, CXCR4 and CD19 (10). The specific role of MKL1 in immune cells is less explored. In human MKL1-deficient neutrophils, many adhesion and actin regulators were downregulated, including *CASS4, ALCAM, MYL9* (11); proteomic studies further identified *WDR* and actin itself. MKL1 also regulated expression of genes involved in interferon signaling, such as *STAT1* and several *IFIT* genes, but MKL1 deficiency also resulted in the upregulation of several genes (11). We have recently shown that in murine dendritic cells, MKL1 deletion did not only impact expression of cytoskeletal genes and genes of cytoskeletal regulators (*Fgr, Hck, Stmn1, Ckap2l, Anln, Tpm2, Tubb5*), but also genes encoding for proteins involved in many other cellular pathways, such as lipid metabolism (12). Therefore, the effect of MKL1 on gene expression in immune cells appears to be cell type-specific. However, it is clear that many MKL/SRF-target genes in leukocytes, as in other cell types, are actin and actin regulators, for example *ACTB*, *WIPF1*, *WDR1*, and *MSN* (13), which regulate actin cytoskeletal dynamics, and therefore affect actin-dependent processes in cells (**Figures 1** and **2**).

## MKL1 Deficiency

Interestingly, patients with MKL1 mutations have recently been reported. The first patient with a homozygous nonsense mutation in MKL1 was reported in 2015 (14), while two more cases (siblings) were reported in 2020 (11). The first sibling died after contracting pneumonia at an early age, the second one underwent a pre-emptive bone marrow transplant. MKL1 mutations result in primary immunodeficiency, with increased susceptibility to bacterial infections (*Pseudomonas* pneumonia). Lymphocyte counts and immunoglobulin levels were normal and no major defect in T- or B-cell function have been reported in these patients (14). Also no major platelet dysfunction has been reported (11, 14). Neutrophil numbers were also normal or elevated because of the concomitant infection. Phagocytosis in patient neutrophils has been reported both as normal – even of larger particles by neutrophils and macrophages (11) – and abnormal (14), while neutrophil reactive oxygen species (ROS) release and bacterial killing have been reported as being intact in MKL1 patients (11). Azurophilic granule release was found to be increased by MKL1-deficient neutrophils under suboptimal stimulation (11).

## Role of MKL/SRF Signaling in Leukocyte Adhesion and Migration

MKL1 deficiency in humans results in a severe migration defect of neutrophils (reduced chemotaxis towards chemotactic agents such as formyl-methionyl-leucyl phenylalanine (fMLF) and complement component 5a (C5a) (11, 14). Also spreading of both neutrophils and dendritic cells was reduced (11, 14). Interestingly, in neutrophils, firm adhesion, and transendothelial migration under shear flow conditions were severely affected, although cell adhesion under static conditions was not reduced (11). Total actin and F-actin levels in neutrophils were reduced (11, 14). Analysis of protein expression levels by high-resolution label-free quantitative mass spectrometry showed that indeed actin and actin regulators (WDR1, ARHGAP9, PFN1) were downregulated in MKL1-deficient neutrophils (11). The dramatic effect of the MKL1 mutation on neutrophil phenotype may be explained by the fact that these cells do not express MKL2, which may compensate for the lack of MKL1 in other cell types, such as fibroblasts. Indeed, primary fibroblasts with MKL1 deficiency display normal migration (11).

The role of MKL1 in dendritic cell adhesion and migration has also been investigated. Interestingly, the MKL1/SRF pathway is downstream of beta2-integrins and beta2-integrin-mediated RhoA activation and F-actin polymerization in murine leukocytes (12). 3D migration of MKL1 knockout dendritic cells in response to CCL19 was normal, while (static) adhesion to integrin ligands was slightly reduced. Integrins function as mechanoreceptors in cells, transmitting mechanical force across the plasma membrane. In MKL1 knockout dendritic cells, integrin-mediated traction forces were reduced. Dendritic cells express both MKL1 and MKL2, which presumably underlies the fact that the adhesion/migration defect of MKL1 knockout dendritic cells was not as severe as in (human) neutrophils, which only express the one isoform.

MKL regulates transcription through interactions with SRF, which has been previously implicated in cell adhesion and migration in immune cells [reviewed in (15)]. SRF-deficient murine macrophages display reduced spreading, migration, and phagocytosis (9). In neutrophils, SRF plays an important role in cell trafficking *in vitro* and *in vivo*. SRF-deficient murine neutrophils failed to traffic into sites of inflammation *in vivo*, and displayed reduced binding to integrin ligands, and cell migration *in vitro* (16). Several actin regulators were downregulated in SRF knockout neutrophils, including *Actb*. Other SRF targets found to be downregulated also in murine MKL1 knockout dendritic cells include *Lima1, Actg1, Cnn2, Tpm4*, and *Wdr1* (12).

MKL1 (and presumably MKL1/SRF-dependent gene transcription of genes encoding cytoskeletal elements and additional proteins involved in signaling, etc) therefore appears to be most important for integrin-mediated cell adhesion under shear flow conditions, where cells have to withstand substantial mechanical forces to resist shear forces in blood vessels. In addition, its role in integrin-mediated traction force generation further implies that MKL1-regulated gene transcription is important for integrin mechanoreceptor function. However, certain other actin-dependent functions appear to function also without MKL1-signaling (ROS release, bacterial killing) and seem intact even in the presence of a strongly reduced G-actin level. It is possible that major actin polymerization is only required for certain highly complex processes related to spreading and motility, whereas other actin-dependent activities such phagocytosis and (intraphagosomal) degranulation for the following killing process of the engulfed microbial particles may be less dependent on complex actin responses. In addition, although there are molecular similarities between the integrin/cytoskeletal processes involved in cell migration and phagocytosis, there are also differences [lack of myosin/contractility in phagocytosis, for example (17)] that may explain the differential dependence on MKL1/SRF of these processes.

Together, these human and murine data point towards an important role for MKL/SRF signaling for regulating myeloid immune cell integrin-mediated adhesion and migration *in vivo*, especially affecting neutrophils. Furthermore, mechanistically MKL1/SRF is probably involved in regulating neutrophil adhesion and migration through regulation of gene transcription of actin and actin-related factors/or other factors that regulate cell adhesion and migration.

## Actin-Related Primary Immunodeficiencies Involving MKL/SRF-Target Genes

Interestingly, several primary immunodeficiencies caused by cytoskeletal abnormalities [“actinopathies” (18–20)] are caused by mutations in genes which are recognized MKL(1/2)-dependent SRF-target genes, namely *ACTB*, *WIPF1*, *WDR1*, and *MSN* (13). Here, we summarize these actinopathies and their effect on immune cell function, in an attempt to dissect which MKL/SRF target genes may be important for leukocyte adhesion and trafficking.

### Cytoplasmic Beta-Actin

Actin is one of the major targets of the MKL/SRF transcription axis (13) and, as observed in MKL1 deficiency (11, 14) important for homeostatic levels of actin. Actin has six isoforms, one of which is cytoplasmic beta-actin (encoded by *ACTB*) (21). Although loss-of-function mutations in *ACTB* have not been reported to cause a PID, it has been described that patients suffer from recurrent infections, which may imply an influence of these mutations on the immune system (22).

One report on immune cell function in a patient with a missense mutation in *ACTB* identified reduced chemotaxis and decreased ROS production of neutrophils (23). This mutation led to the expression of both normal and mutated cytoplasmic beta-actin in patient cells, including fibroblasts, leukocytes, Epstein–Barr virus-transformed B-cells, and platelets (23). Further investigation revealed that this mutated beta-actin had a slower polymerization rate than normal actin (24). More recently, a lack of beta-actin in mouse embryonic fibroblasts was shown to downregulate interferon-stimulated genes due to instability of the transcription factor interferon regulatory factor 3, thereby impairing the antiviral response (25). Likewise, SRF has been previously shown to be important for type I interferon signaling by induction of several interferon-stimulated genes (26).

### Wiskott-Aldrich Syndrome Protein-Interacting Protein

Actin polymerization, i.e., the assembly of G-actin into F-actin, is mediated by the Wiskott-Aldrich syndrome protein (WASP), as it activates the actin-related proteins-2/3 (ARP2/3) complex. This complex, consisting of seven subunits, is an actin nucleator which initiates the branching of actin filaments (27). The Wiskott-Aldrich syndrome protein-interacting protein (WIP) interacts with WASP, thereby stabilizing its inactive state. When WASP gets activated, WIP is phosphorylated and dissociates from WASP (28, 29). The human gene encoding for WIP, *WIPF1*, has been identified to be a MKL-dependent SRF-target gene (13).

Only a handful of patients with WIP deficiency, caused by autosomal recessive mutations in *WIPF1*, have been identified thus far (30–32). As WIP regulates the stability and localization of WASP (33), loss of WIP will also lead to absence of WASP (30, 34). Therefore, patients with WIP deficiency have a similar clinical phenotype as WAS patients, i.e., recurrent infections, thrombocytopenia, and eczema (31, 35).

The adaptive immune cells, i.e., T-cells and B-cells, are most affected in WIP deficiency. Patients suffer from T-cell lymphopenia, where CD8^+^ T-cells are more affected (35). Functionally, T-cells showed impaired proliferation, defective chemotaxis, defective exocytosis, and reduced target killing, most likely because there is a failure to assemble the immunological synapse (30, 32). Also, T-cells had an abnormal morphology, and failed to elongate and assemble a leading- and trailing edge upon stimulation (32). Natural killer (NK) cell–mediated cytotoxicity was reported to be reduced (30), and B-cells showed a chemotaxis defect, which could be rescued when WIP expression was restored in these cells (32). No defects in neutrophil function were reported in WIP patients, and monocyte-derived dendritic cells showed a normal phenotype (32). As the phenotype is quite different from that seen in MKL1 deficiency, *WIPF1* may not be the most important gene regulating neutrophil trafficking downstream of MKL/SRF.

### WD Repeat-Containing Protein 1

Actin-interacting protein 1 (Aip1), encoded *WDR1*, is an actin-binding protein which enhances the disassembly and severing of actin filaments by cofilin (36, 37). *WDR1* is identified as an MKL/SRF target gene (13), and expression of *WDR1* is strongly reduced in murine cells lacking *Srf* (38), in murine dendritic cells lacking *Mkl1* (12), and on the protein level in neutrophils of MKL1-deficient patients (11). Mutations in *WDR1* have been reported to cause immunodeficiency (39–41), and *WDR1* is considered to be the main candidate gene to cause the “Lazy Leukocyte Syndrome”, first described in 1971 (42). Patients with loss-of-function mutations in *WDR1* are reported to suffer from recurrent infections, mild neutropenia and impaired wound healing (39), but also a separate syndrome of auto-inflammation, periodic fever, and thrombocytopenia has been reported (40, 41).

WDR1-deficient patient cells, including neutrophils, monocytes, dendritic cells, and lymphocytes, are defective in actin depolymerization, resulting in increased F-actin levels (39–41). About 50% of the neutrophils had an altered herniation of their nuclear lobes, which co-localized with increased F-actin staining (39, 40). Neutrophils had a severe chemotaxis defect and showed abnormal spreading on glass, while *Staphylococcal aureus* killing and phagocytosis of opsonized *Escherichia coli* were reported to be normal. ROS production was found both normal and enhanced in WDR1-deficient neutrophils (39–41). Monocytes showed increased spreading over fibronectin, and CD14^+^ peripheral blood mononuclear cells showed an increased caspase-1 activation, which corresponds with auto-inflammation (40, 41).

Furthermore, defects in the adaptive immune system were observed. T-cells had an increased spreading capacity and mildly impaired proliferation, but normal T-cell receptor internalization, normal migration, and normal killing of target cells by CD8^+^ T-cells (40). B-cells showed increased apoptosis on B-cell receptor/Toll-like receptor stimulation and abnormal spreading, but normal migration. Also, patients suffered from peripheral B-cell lymphopenia and there was a lack of switched memory B-cells, reduced clonal diversity and paucity of B-cell progenitors in the bone marrow (40).

### Moesin

Moesin (encoded by *MSN*) is a member of the ezrin, radixin, moesin (ERM) protein family, which link cortical actin filaments to the plasma membrane and membrane receptors. ERM proteins are important for structural stability and the integrity of the cell cortex (43). *MSN* is recognized to be a MKL-dependent SRF-target gene (13). As *MSN* is located on the X chromosome, the immunodeficiency was termed X-linked moesin associated immunodeficiency (44). Patients suffer from recurrent bacterial- and viral infections, persistent eczema and lymphopenia, as well as fluctuating neutropenia (44–46).

MSN-deficient T-cells showed impaired proliferation, impaired chemotaxis, and increased adhesion to vascular cell adhesion molecule 1 (44). While no functional analysis was done on neutrophils or monocytes from these patients, neutrophils from male moesin-deficient mice (*Msn* ^-/Y^) did display elevated rolling velocity in inflamed blood vessels, indicating that moesin has an important role in slow leukocyte rolling and subsequent trans-endothelial migration (47). Also, moesin-deficient mice showed a reduced neutrophil microbial killing ability towards *Pseudomonas aeruginosa* and reduced neutrophil-mediated vascular inflammation, while neutrophil adhesion was not affected (48).

### Actin-Related Proteins 2/3 Complex Subunit 1B

The ARP2/3 complex, consisting of seven subunits, plays an essential role in the formation of branched actin networks by nucleating a daughter filament to the side of a pre-existing actin filament (27). These branched actin networks are especially of importance for generating a protrusive force that aids in cellular adhesion and motility (49). Almost all subunits of the ARP2/3 complex have been recognized to be MKL/SRF-target genes [ARP2, ARP3, ARP2/3 complex subunit 2 (ARPC2), ARPC4, and ARPC5] (13), suggesting that MKL1/SRF is involved in actin branching by regulating transcription of ARP2/3 complex family members. ARPC1 is present in two isoforms in humans, ARPC1A and ARPC1B, the latter being the dominant form in hematopoietic cells (50, 51). Although it is not (yet) identified as a MKL/SRF-target gene, patients with ARPC1B deficiency have a very similar neutrophil phenotype compared to MKL1-deficient patients.

Patients with ARPC1B deficiency suffer from a combined immunodeficiency including a neutrophil defect, resulting clinically in bacterial- and viral infections, bleeding tendency, eczema, allergy, and vasculitis (50–55). Similar to MKL1 deficiency, neutrophils have a severe migration defect due to an actin polymerization defect. Also, ROS production and the phagocytosis and killing of bacteria were found to be intact, and azurophilic granule release was increased under suboptimal stimulation (50).

Although no major defects in T- or B-cell function have been reported in MKL1 deficiency, ARPC1B deficiency does result in lymphocytes defects, including defective migration and proliferation, defective immunological synapse assembly by T-cells (53, 55), impaired regulatory T-cell suppressor activity and impaired NK-cell degranulation (52). Primary fibroblasts showed normal migration, most likely due to expression of ARPC1A in these cells (similar to possible MKL2 compensation in MKL1 deficiency) (50).

Thus, MKL1-deficient neutrophils have an almost identical phenotype as ARPC1B-deficient neutrophils, which could be explained by the involvement of MKL/SRF in transcription of ARP2/3 complex family members. However, ARPC1B deficiency also affects lymphocytes, which has not been observed in MKL1 deficiency. A possible explanation might be redundancy of MKL2 in lymphocytes, which express low levels of this isoform.

In conclusion, analysis of primary immunodeficiencies involving MKL/SRF targets and putative targets (**Table 1**) implicate ARP-subunits as possible targets downstream of MKL/SRF that could be involved in regulating leukocyte adhesion and migration *in vivo.*

**Table 1:** List of MKL/SRF-related actinopathies with corresponding protein function, clinical symptoms, and reported functionally affected hematopoietic cells in these patients.

Actinopathy (gene)	Protein function	MKL/SRF target gene	Clinical symptoms	Hematopoietic cells functionally affected in patients	References
MKL1 deficiency (*MKL1*)	Coactivator of transcription factor SRF, thereby involved in regulating cytoskeletal gene transcription	Yes	Severe, recurrent bacterial infections	Neutrophils, dendritic cells	(11, 14)
Cytoplasmic beta-actin mutations (*ACTB*)	Non-muscle actin; one of the six isoforms of actin	Yes	Recurrent infections	Neutrophils	(23)
WIP deficiency (*WIPF1*)	Interacts with WASP, thereby stabilizing its inactive state	Yes	Recurrent infections, thrombocytopenia, eczema	NK-cells, T-cells, B-cells	(30–32, 35)
WDR1 deficiency (*WDR1*)	Actin-binding protein, enhances the disassembly and severing of actin filaments by cofilin	Yes	Recurrent infections, mild neutropenia, impaired wound healing, and auto-inflammation, periodic fever, thrombocytopenia	Neutrophils, monocytes, dendritic cells, T-cells, B-cells	(39–41)
Moesin deficiency (*MSN*)	Member of the ERM protein family; links cortical actin filaments to the plasma membrane and membrane receptors	Yes	Recurrent bacterial- and viral infections, eczema, lymphopenia, fluctuating neutropenia	T-cells	(44–46)
ARPC1B deficiency (*ARPC1B*)	Subunit of the ARP2/3 complex, thereby involved in branching of actin filaments	No, but most other ARP2/3 subunits are MKL/SRF-target genes	Recurrent bacterial- and viral infections, bleeding tendency, eczema, allergy, vasculitis	Neutrophils, NK-cells, T-cells, B-cells, platelets	(50–55)

A more elaborate table of immunological actinopathies, including those caused by mutations in non-MKL/SRF target genes, can be found in the review by Sprenkeler et al. (56). ARPC1B, actin-related protein complex 2/3 subunit 1B; ARP2/3 complex, actin-related protein complex 2/3; ERM, ezrin/radixin/moesin; MKL1, megakaryoblastic leukemia 1; NK-cells, natural killer cells; SRF, serum response factor; WASP, Wiskott-Aldrich syndrome protein; WDR1, WD repeat-containing domain 1; WIP, WASP-interacting protein.

## Targeting of MKL/SRF in Immune Cell-Mediated Inflammation—New Possibilities?

MKL/SRF signaling and MKL/SRF regulated gene expression of actin-related factors are important in leukocyte adhesion and migration, as shown by the actin-related primary immunodeficiencies described above. Interestingly, genetic syndromes also exist where actin dynamics is upregulated, eg X-linked neutropenia. In this disease, neutrophil levels in blood are low, but there is increased migration of leukocytes into tissues. X-linked neutropenia is caused by gain-of-function mutations in *WASP*, leading to increased actin dynamics, and, as a consequence, to upregulation of actin-dependent neutrophil adhesion, migration, and recruitment into tissues (57).

As MKL/SRF signaling and downstream targets of this pathway are clearly associated with leukocyte migration, targeting this pathway in inflammatory diseases where leukocyte migration into tissues is upregulated may offer a new therapeutic strategy. MKL/SRF signaling is important in many cell types and tissues, and is also implicated in disease in different settings. Indeed, the MKL/SRF pathway has already been targeted in a number of different diseases, including several with an immune/inflammatory cell component, such as fibrosis and corneal wound healing. Interestingly, MKL/SRF inhibitors have been used to treat fibrosis in lung, skin, colon, liver, joints, and the eye (both the external cornea and the internal subretina) (**Table 2**). The target for these therapeutic approaches are myofibroblasts and the excess extracellular matrix (ECM) production by these cells, as MKL1 has been found to be important for myofibroblast differentiation (74). However, in scleroderma/systemic sclerosis patient skin samples, a disease associated with internal organ stiffening due to fibrosis, nuclear MKL1 has also been found in infiltrating macrophages (65), where it has been linked to proinflammatory gene expression (75, 76), indicating that also macrophages could be a target cell type for SRF inhibition in fibrosis.

**Table 2:** MKL/SRF targeting in disease.

Disease	Inhibitor	Effect	Model	References
Fibrosis (Eye; subconjunctival scarring after glaucoma filtration surgery)	CCG-222740 and CCG-203971	Daily administration of 100 mg/kg CCG-222740 reduced and delayed subconjunctival scarring after glaucoma filtration surgery and improved surgery success by 67%. CCG-203971 delayed slightly less subconjunctival scarring and improved surgery success by 33% compared to a control. Inhibitors decreased cellularity and α-smooth muscle actin (α-SMA expression	Fibroblast mediated collagen contraction assay (inhibitor tests); *in vivo* scar tissue formation (subconjunctival scarring after glaucoma filtration surgery) model in rabbits	(58)
Subretinal fibrosis	CCG-1423	Inhibited cell migration; inhibition of TGF-β induced MKL1 shuttling, Paxilin and pro-MMP-2 expression; injection into vitreous cavity inhibited fibrosis development	Human retinal pigment epithelial cells (RPE-1 cells); murine *in vivo* CNV model	(59)
Dermal fibrosis	CCG-203971	Inhibited expression of connective tissue growth factor (CTGF), α-SMA, and collagen 1 (COL1A2) in fibroblasts. In mice CCG-203971 prevented bleomycin-induced skin thickening and collagen deposition	Systemic sclerosis fibroblasts, Bleomycin-Induced Injury Model	(60)
Dermal scarring	Fasudil (ROCK inhibitor)	Inhibited fibroblast contractility, and myofibroblast formation *in vitro*; inhibited wound contraction *in vivo*	Wistar-han rats	(61)
Colon fibrosis	CCG-1423, CCG-100602 and CCG-203971	Repressed matrix-stiffness and TGF-β mediated fibrogenesis	*In vitro* models using human colonic fibroblast CCD-18co cells (CRL-1459, derived from a female donor)	(62)
Liver fibrosis	CCG-1423	Alleviated c-Abl inhibition. c-Abl activation is associated with cellular fibrogenesis	Primary hepatic stellate cells, Immortalized human hepatic stellate cells (LX-2)	(63)
Osteoarthritis-related fibrosis	CCG-100602, Y-27632 (ROCK inhibitor)	Suppressed mRNA levels of α-SMA and type I collagen	Fibroblast-like synoviocytes cell line	(64)
Scleroderma/systemic sclerosis (lung and skin in study)	CCG-1423	Decreases collagen, α-SMA, and CCN2 expression in SSc cells	Sclerodermal fibroblasts	(65)
Pulmonary fibrosis	CCG-203971 Fas-activating antibody	*In vitro:* inhibited MKL1 shuttling, blocked myofibroblast differentiation, inhibited TGF-β1-induced expression of fibronectin, X-linked inhibitor of apoptosis, and plasminogen activator inhibitor-1. *In vivo*: reduced lung collagen content, decreased alveolar plasminogen activator inhibitor-1 and promoted myofibroblast apoptosis	Adult primary human lung and primary human fetal lung fibroblast cell lines (CCL-210 IMR-90) Murine models of fibrosis induced by: •bleomycin •targeted type II alveolar epithelial injury	(66)
Pulmonary fibrosis	CCG-1423	Inhibited TGF-β–induced α-SMA expression	*In vitro* assay with primary isolated human lung fibroblasts	(67)
Cardiomyopathy	CCG-1423-8u (SRF targeting)	Inhibition of SRF in a disease mouse model increased survival from 98 days (ctrl) to 116	Murine CAP2 KO *in vivo* model	(68)
Vascular proliferative diseases	CCG-1423	Intraperitoneal treatment with 0.15 mg/kg for 3 weeks inhibited progression of vascular remodeling in arteries	Mice subjected to femoral artery wire injury	(69)
Aortic stiffening (cardivascular morbidity)	CCG-100602 SRF/myocardin inhibitor	Reduced the stiffness of reconstituted tissue and changed LOX gene expression	Primary vascular smooth muscle cells (VSMCs) isolated from rats used for other experiments in the study, *in vitro* reconstituted tissue	(70)
Aortic stiffening	CCG-100602 1.5 mg/kg/day for 2 weeks delivered by subcutaneous osmotic minipump	Reduced aortic stiffness and then blood pressure in SHR but not in WKY rats	Spontaneously hypertensive rats (SHR) Normotensive Wistar-Kyoto (WKY) rats	(71)
Hypertension	Y-27632 (Rock inhibitor), CCG-100602	Reduced aorta wall stiffness and blood pressure *in vivo*	*In vitro* VSMC models and SHR and WKY rats	(72)
Pulmonary Arterial Hypertension	CCG-1423	Attenuated pulmonary arterial hypertension and lung vascular remodeling	Sugen/hypoxia rats	(73)

Targeted diseases, inhibitors used, and the model systems used are described in this table.

Targeting the MKL/SRF pathway to improve corneal wound healing has emerged as an attractive strategy. The cornea is a transparent endothelial cell layer in the eye and its thinning as well as replacement with connective and corneal stroma is associated with blindness. In the cornea, stromal cells produce cytokines like interleukin-1 (IL-1) and transforming growth factor-β (TGF-β) to induce inflammatory and degradative processes or to induce ECM deposition (77). TGF-β1 activates the MKL/SRF pathway (67, 78), indicating MKL/SRF could play a role in corneal wound healing (79). Indeed, eye drops supplemented with the inhibitor Y-27632 to inhibit Rho Associated Kinase (ROCK) upstream of MKL/SRF, have been used to improve corneal endothelial wound healing *in vivo* (80).

## Summary and Future Prospects

MKL1 deficiency is one of the most recently identified primary immunodeficiencies and is associated with globally impaired actin regulation, defective cell adhesion and abnormal trafficking of myeloid leukocytes. Studies in both mouse and man have provided novel insights into its complex role in immune cell migration and function in the context of infections. The main cell types affected by MKL1 deficiency and MKL/SRF signaling are neutrophils and macrophages. Neutrophils are involved in a wide variety of inflammatory processes including acute organ injury, cystic fibrosis, ischemia reperfusion injury, atherosclerosis, and autoimmunity (such as rheumatoid arthritis). It is tempting to speculate that MKL/SRF, which is already being targeted in several diseases (**Table 2**) could also be used to reduce neutrophil and macrophage trafficking in these and other inflammatory disorders.

## Author Contributions

ES, CG, TK, and SF wrote the manuscript on behalf of the LADOMICS consortium. IF analyzed bioinformatics data and made **Figure 2**. All authors contributed to the article and approved the submitted version.

## Funding

The work in the authors’ laboratories is supported by the Academy of Finland, Svenska Kulturfonden, Liv och Hälsa, HiLIFE, and E-Rare/Academy of Finland (LADOMICS) (to SF), Magnus Ehrnrooth foundation (to CG). TK and ES were partially funded by the European Union’s Horizon 2020 research and innovation programme under Grant Agreement No.668303, Program on Prevention Outcomes Practices Grant PPOP-12-001, the Center of Immunodeficiencies Amsterdam Grant CIDA-2015, and the E-Rare ZonMW grant #90030376506.

## Conflict of Interest

The authors declare that the research was conducted in the absence of any commercial or financial relationships that could be construed as a potential conflict of interest.
